# Meta-Analysis: Effects of Probiotic Supplementation on Lipid Profiles in Normal to Mildly Hypercholesterolemic Individuals

**DOI:** 10.1371/journal.pone.0139795

**Published:** 2015-10-16

**Authors:** Mikiko Shimizu, Masayuki Hashiguchi, Tsuyoshi Shiga, Hiro-omi Tamura, Mayumi Mochizuki

**Affiliations:** 1 Department of Hygienic Chemistry, Faculty of Pharmacy, Keio University, 1-50-30 Shibakoen, Tokyo, 105–8512, Japan; 2 Division for Evaluation and Analysis of Drug Information, Faculty of Pharmacy, Keio University, 1-50-30 Shibakoen, Tokyo, 105–8512, Japan; 3 Department of Cardiology, Tokyo Women’s Medical University, 8–1 Kawada-cho, Shinjuku-ku, Tokyo, 162–8666, Japan; University College Dublin, IRELAND

## Abstract

**Introduction:**

Recent experimental and clinical studies have suggested that probiotic supplementation has beneficial effects on serum lipid profiles. However, there are conflicting results on the efficacy of probiotic preparations in reducing serum cholesterol.

**Objective:**

To evaluate the effects of probiotics on human serum lipid levels, we conducted a meta-analysis of interventional studies.

**Methods:**

Eligible reports were obtained by searches of electronic databases. We included randomized, controlled clinical trials comparing probiotic supplementation with placebo or no treatment (control). Statistical analysis was performed with Review Manager 5.3.3. Subanalyses were also performed.

**Results:**

Eleven of 33 randomized clinical trials retrieved were eligible for inclusion in the meta-analysis. No participant had received any cholesterol-lowering agent. Probiotic interventions (including fermented milk products and probiotics) produced changes in total cholesterol (TC) (mean difference –0.17 mmol/L, 95% CI: –0.27 to –0.07 mmol/L) and low-density lipoprotein cholesterol (LDL-C) (mean difference –0.22 mmol/L, 95% CI: –0.30 to –0.13 mmol/L). High-density lipoprotein cholesterol and triglyceride levels did not differ significantly between probiotic and control groups. In subanalysis, long-term (>4-week) probiotic intervention was statistically more effective in decreasing TC and LDL-C than short-term (≤4-week) intervention. The decreases in TC and LDL-C levels with probiotic intervention were greater in mildly hypercholesterolemic than in normocholesterolemic individuals. Both fermented milk product and probiotic preparations decreased TC and LDL-C levels. Gaio and the *Lactobacillus acidophilus* strain reduced TC and LDL-C levels to a greater extent than other bacterial strains.

**Conclusions:**

In conclusion, this meta-analysis showed that probiotic supplementation could be useful in the primary prevention of hypercholesterolemia and may lead to reductions in risk factors for cardiovascular disease.

## Introduction

Hypercholesterolemia is a major risk factor for lifestyle-related disease such as atherosclerosis and cardiovascular disease (CVD), including coronary heart disease (CHD) and stroke [1]. Epidemiological and clinical investigations of statins have demonstrated that elevated serum and dietary cholesterol levels are associated with increased risk of CHD [2–4].

In recent years, there has been increasing interest in alternative therapies to lower serum cholesterol, particularly when conventional drug therapy is considered unsuitable, whether on the grounds of cost, safety, or simply personal preference. Probiotic products are widely available on the market in the USA, Europe, and Japan and are defined as live microbial supplements that colonize the gut while providing benefits to the host [5].

Several experimental and clinical studies previously found that probiotic bacteria such as *Lactobacillus* and *Bifidobacterium* have beneficial effects on serum lipid profiles [6–16]. *In vitro* studies suggested that certain strains of lactobacilli could remove cholesterol [6, 7]. Similarly, the results of limited animal [8–10] and clinical studies [11–16] suggested that probiotic bacteria of the lactic acid bacteria group and *Bifidobacterium* may have potential serum cholesterol-regulating properties. However, the number of participants enrolled in those trials was too small to achieve statistically conclusive results. In addition, two reports indicated the effect of probiotics on plasma cholesterol using the meta-analysis approach [17, 18]. However, one of those reports (by Agerholm-Larsen et al. [17]) evaluated only the effects of Gaio, which is a yoghurt product fermented with Causido, composed of one strain of *Enterococcus faecium* and two strains of *Streptococcus thermophilus*, and that meta-analysis suggested that it had cholesterol-lowering effects. The other meta-analysis indicated a decrease in serum lipid concentrations with the intake of probiotics based on studies of various bacterial strains [18]. However, there are no detailed analyses of the factors related to decreased serum lipid concentrations with probiotic intake including characteristics of study participants, duration of intake, probiotic preparation, etc. The combined results of the numerous previous studies including various factors show bias when subjected to meta-analysis. It would also be useful to clarify the factors involved in the decreased lipid concentrations with probiotic intake in a stratified analysis. Therefore, the objective of this investigation was to clarify comprehensively the effects of probiotic supplementation on serum lipid profiles in a meta-analysis, including subanalyses of the factors that might have affected previous results.

## Methods

### Search strategy

The electronic databases Embase (1974 to July 2014), PubMed (1964 to July 2014), Cochrane Central Register of Controlled Trials (Issue 7 of 12, July 2014), and Igaku Chuo Zasshi (1983–July 2014) were used for systematic literature searches. As subject headings, we employed free-language terms for hypercholesterolemia combined with each of the following: “probiotics”; “yogurt”; “yoghurt”; “lactic acid bacterium”; “*Lactobacillus*”; “*Bifidobacterium*”; “*Enterococcus*”; “*Streptococcus*”; “*Saccharomyces*”; and “*Lactococcus*”. The type of article was limited to randomized controlled trials (RCTs) or clinical trials. Only Japanese and English articles were searched. A systematic literature search was performed on Embase, PubMed, Cochrane Central Register of Controlled Trials, and Igaku Chuo Zasshi for reports that were published through July 30, 2014.

### Inclusion criteria

Two investigators (M.S., M.H.) examined the title and abstract of each paper and then the full paper if necessary. The selection criteria were (1) the participants were healthy or hypercholesterolemic individuals; (2) the reports were of RCTs studying the consumption of probiotics and measurement of serum lipid concentrations, i.e., total cholesterol (TC), low-density lipoprotein cholesterol (LDL-C), high-density lipoprotein cholesterol (HDL-C), and triglycerides (TGs); and (3) all ages from infants to the elderly were included.

### Data extraction

Standardized data abstraction sheets were prepared. Data were extracted for the clinical study design used, type and duration of probiotic treatment, characteristics of enrolled participants (physical condition, number, gender, age, body mass index [BMI]), and key outcome data such as serum lipid levels including TC, LDL-C, HDL-C, and TGs. We aimed to analyze summary data (mean values ± standard deviation) on TC, LDL-C, HDL-C, and TGs. Before meta-analysis was performed, the lipid levels in milligrams per deciliter were converted into millimoles per liter prior to computations. The conversion factors were 1 mg/dL = 0.0259 mmol/L for cholesterol and 1 mg/dL = 0.0113 mmol/L for TGs.

We extracted data presented in the original papers. All articles were examined independently for eligibility by two reviewers (M.S., M.H.). Disagreements were resolved by consulting a third reviewer (M.M.). Quality was assessed using the Jadad score system [19] based on the three items of randomization, double blinding, and description of withdrawals and dropouts to generate scores from 0 to 5. The selected studies were scored independently by two investigators, and if there were disagreements, they consulted a third reviewer to obtain the final scores. The quality of papers was considered unacceptable for inclusion if a score lower than 3 was assigned.

### Statistical analysis

All statistical analyses were performed using Review Manager (RevMan) 5.3.3 software (Cochrane Collaboration, 2014). The values used for the meta-analysis were the differences calculated for TC, LDL-C, HDL-C, and TGs as: (post–pre)_intervention_−(post–pre)_control_. In other words, the difference was the change in the intervention group (I) minus the change in the control group (C). The effects were analyzed based on a fixed-effects model using the method of Mantel-Haenszel on a per-protocol basis. Heterogeneity between studies was assessed using the chi-square test and I^2^ statistic with values ranging from 0% to 100%. An I^2^ value of less than 30% indicated no obvious heterogeneity, and a value greater than 50% suggested increasing heterogeneity. Statistical significance for the test of heterogeneity was set at 0.05. If significant heterogeneity existed, it would have been inappropriate to combine the data for further analysis using a fixed-effects model. In such cases, the random-effect model was used for calculation.

Subanalyses for the meta-analysis were planned depending on pre TC and pre LDL-C levels, BMI, age, duration of intake of probiotics, probiotic preparations, and bacterial strains. For these subanalyses, we used the summary statistics that were available within studies. Finally, we used funnel plot asymmetry to detect any publication bias in the meta-analysis, and Begg’s rank correlation method and the Egger weighted regression method were applied to measure funnel plot asymmetry.

## Results

### Description of studies

**Fig 1:**
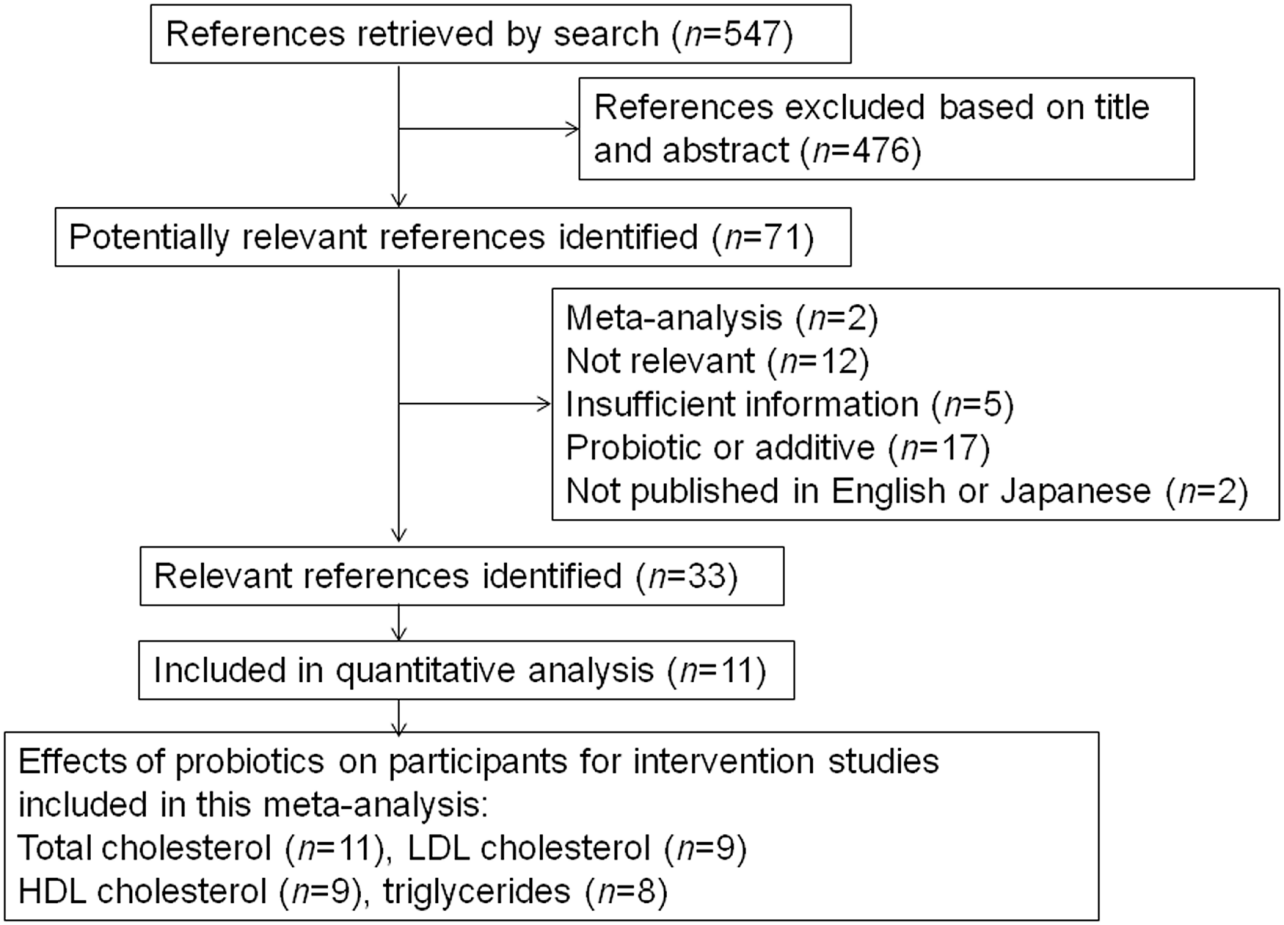
Flow diagram of the selection of published articles on probiotics for the treatment of hyperlipidemia for inclusion in this meta-analysis.

A flow diagram of article selection for this meta-analysis is shown in Fig 1. Five hundred forty-seven articles were initially retrieved from our web search. After examining the titles and abstracts, and then the full papers if necessary, 33 articles met the inclusion criteria.

Secondarily, we selected 11 [13, 15, 20–28] articles that described data on differences before and after intervention in serum lipids including TC, LDL-C, HDL-C, and TGs. The characteristics of these 11 articles are summarized in Table 1. Of these selected articles, five used a double-blind design, one a triple-blind design, and two a single-blind design with a placebo. Four studies used a cross-over design with two phases [13, 15, 22, 26]. One study, by Bertolami et al. [22], used a cross-over design with three phases: phase 1, diet only; phase 2, diet + placebo; and phase 3, diet + Gaio. We used phase 3 –phase 2 as the treatment measure, and phase 2 –phase 1 as the control measure in our analyses. Although seven articles were based on studies of patients with primary or mild hypercholesterolemia, no participant had received any cholesterol-lowering agent. These 11 articles consisted of 8 [13, 15, 20–22, 24, 26, 27] using fermented milk products and 3 [23, 25, 28] using probiotic capsules as test drugs. One of 3 articles [23] included both probiotics and prebiotics, i.e., a fructo-oligosaccharide in a rice starch base, as test drugs.

**Table 1:** Characteristics of the 11 studies included in the meta-analysis.

Reference	Design	Participants	Group	Gender(M/F)	BMI(kg/m^2^) (SD)	Age(y) (SD)	Intake of probiotic product	Duration(weeks)	Jadadscore
Agerbaek [20]	RCT, double blind	Healthy, nonobese, normocholesterolaemic individuals	C	28/0	24.1 (1.7)	44	Placebo product (chemically fermented), 200 mL daily	3, 6	5
		(5.0 ≤TC ≤6.5 mmol/L, TG <5 mmol/L, BMI <27.5 kg/m^2^)	I	29/0	24.3 (2.0)		Gaio (*E*. *faecium*: ∼2×10^8^ CFU/mL, *S*. *thermophilus*: ∼7×10^8^ CFU/mL), 200 mL daily		
Agerholm-Larsen [21]	RCT, double blind	Healthy (no diabetes, kidney, or liver disease), weight-stable, overweight individuals (25.0 < BMI <37.5 kg/m2)	C (PP)	3/7	29.9 (3.5)	38 (10)	PP	4, 8	5
			C (PY)	5/9	30.0 (3.4)	39 (8)	PY, 450 mL daily		
			I (*StLa*)	4/12	30.0 (2.8)	39 (8)	*StLa (S*. *thermophilus*: ∼10×10^7^ CFU/mL, *L*. *acidophilus*: ∼2×10^7^ CFU/mL), 450 mL daily		
			I (*StLr*)	4/10	30.2 (2.6)	38 (9)	St*Lr* (S. thermophilus: ∼8×10^8^ CFU/mL, *L*. *rhamnosus*: ∼2×10^8^ CFU/mL), 450 mL daily		
			I (Gaio)	4/12	30.1 (2.4)	38 (8)	Gaio (*E*. *faecium*: ∼6×10^7^ CFU/mL, *S*. *thermophilus*: ∼1×10^9^ CFU/mL), 450 mL daily		
Anderson [13]	RCT, single blind, cross-over	Individuals with primary hypercholesterolemia (type IIa or Iib lipoprotein hypercholesterolemia), diabetes mellitus, or hypothyroidism	C	9 / 10		58 (13)	Placebo fermented milk product (*S*. *thermophilus* MUH34 culture), 200 g daily	2, 3, 4	3
			I	9 / 12		55 (14)	Fermented milk product *(L*. *acidophilus* L1 (1×10^7^ CFU/g ≤), 200 g daily		
Ataie-Jafari A [15]	RCT, single blind, cross-over	Patients with mild-to-moderate hypercholesterolemia without coronary heart disease, diabetes, or hypothyroidism (5.17 < TC < 7.76 mmol/L, BMI ≥ 30 kg/m^2^)	C	4/10	26.1 (2.9)	50.5 (6.8)	Traditional yogurt *(S*. *thermophilus*, *L*. *delbrueckii* subsp. *bulgaricus*), 300 g daily	6	3
			I	4/10			Probiotic yoghurt (*L*. *acidophilus* ≤1×10^6^ CFU/g, *B*. *lactis* ≤1×10^6^ CFU/g), 300 g daily		
Bertolami [22]	RCT, double blind, cross-over	Individuals with primary hypercholesterolemia without diabetes, hypothyroidism, nephrotic syndrome, or obesity (3.36 <LDL-C <5.69 mmol/L, TG <3.95 mmol/L, BMI <30 kg/m^2^)	C	11/21	≤30	56 (8)	PY (chemically fermented), 200 g daily	8	5
			I	11/21			Gaio (*E*. *faecium*: 10^5^–10^9^ CFU/mL, *S*. *thermophilus*: 5–20×10^8^ CFU/mL), 200 g daily		
Greany [23]	RCT, single blind	Healthy men and premenopausal women (18 ≤BMI ≤30 kg/m^2^)	C	7 / 11	22.8 (3.5)	26.7 (5.2)	PP (rice starch)	8	3
			I	15 / 22	24.1 (3.1)	26.8 (5.0)	Probiotic capsules (*L*. *acidophilus* strain DDS–1 (1.25×10^9^ CFU), *B*. *longum* strain UABL–14 (1.25×10^9^ CFU), 10–15 mg fructo-oligosaccharide in a rice starch base)		
Hata [24]	RCT	Hypertensive outpatients without secondary causes of hypertension	C	4 / 9	21.9 (2.5)	73 (38)	Placebo drink,100 mL daily	8	3
			I	4 / 13	19.1 (2.9)	77 (31)	Sour milk drink *(L*. *helveticus (≒7*.*0×10* ^*11*^ *CFU/L)*, *S*. *cerevisiae (≒2*.*5×10* ^*9*^ *CFU/L)*, 100 mL daily		
Simons [25]	RCT, double blind	Individuals with elevated serum cholesterol (TC ≥4.0 mmol/L, TG ≤4 mmol/L)	C	8 / 13	24.4 (3.7)	53 (11)	PP, 2 capsules twice daily	10	5
			I	8 / 15	27.0 (5.7)	50 (12)	PCC *L*. *fermentum* (2×10^9^ CFU), 2 capsules twice daily		
St-Onge [26]	RCT, single blind cross-over	Mildly hypercholesterolemic men (6 ≤TC ≤10 mmoL)	C	13 / 0	30.2 (4.4)	47 (9)	Milk, 500 mL daily	4	3
			I	13 / 0			Kefir, 500 mL daily		
Sadzadeh-Yeganeh [27]	RCT, triple blind	Women (TC <6.2 mmoL/L, TG <2.3 mmoL/L, BMI <30.0 kg/m^2^)	C	0 / 30	23.8 (3.0)	34.7	No yoghurt	6	5
			I	0 / 30	24.0 (2.4)	35.5	Probiotic yoghurt (*L*. *bulgaricus*, *S*. *thermophilius*, *B*. *lactis* Bb12 (3.7×10^7^ CFU), *L*. *acidophilus* La5 (3.9×10^7^ CFU), 300 g daily		
Jones [28]	RCT, double blind	Healthy hypercholesterolemic individuals (LDL-C <3.4 mmol/L, TG <4.0 mmol/L, BMI 22–32 kg/m^2^)	C	27 / 34	27.6 (2.8)	47.6 (12.9)	PP, 1 capsule twice daily	6, 9	5
			I	28 / 39	26.8 (3.1)	50.5 (14.0)	*L*. *reuteri* NCIMB 30242 (2×10^9^ CFU/capsule), 1 capsule twice daily		

C: control (placebo); I: intervention; PP: placebo pill; PY: placebo yogurt; RCT; randomized controlled trial; TC: total cholesterol; LDL-C: low-density lipoprotein cholesterol; *StLa*: *Streptococcus thermophilus* and *Lactobacillus acidophilus*; *StLr*: *Streptococcus thermophilus* and *Lactobacillus rhamnosus*

### Outcomes of the meta-analysis

**Fig 2:**
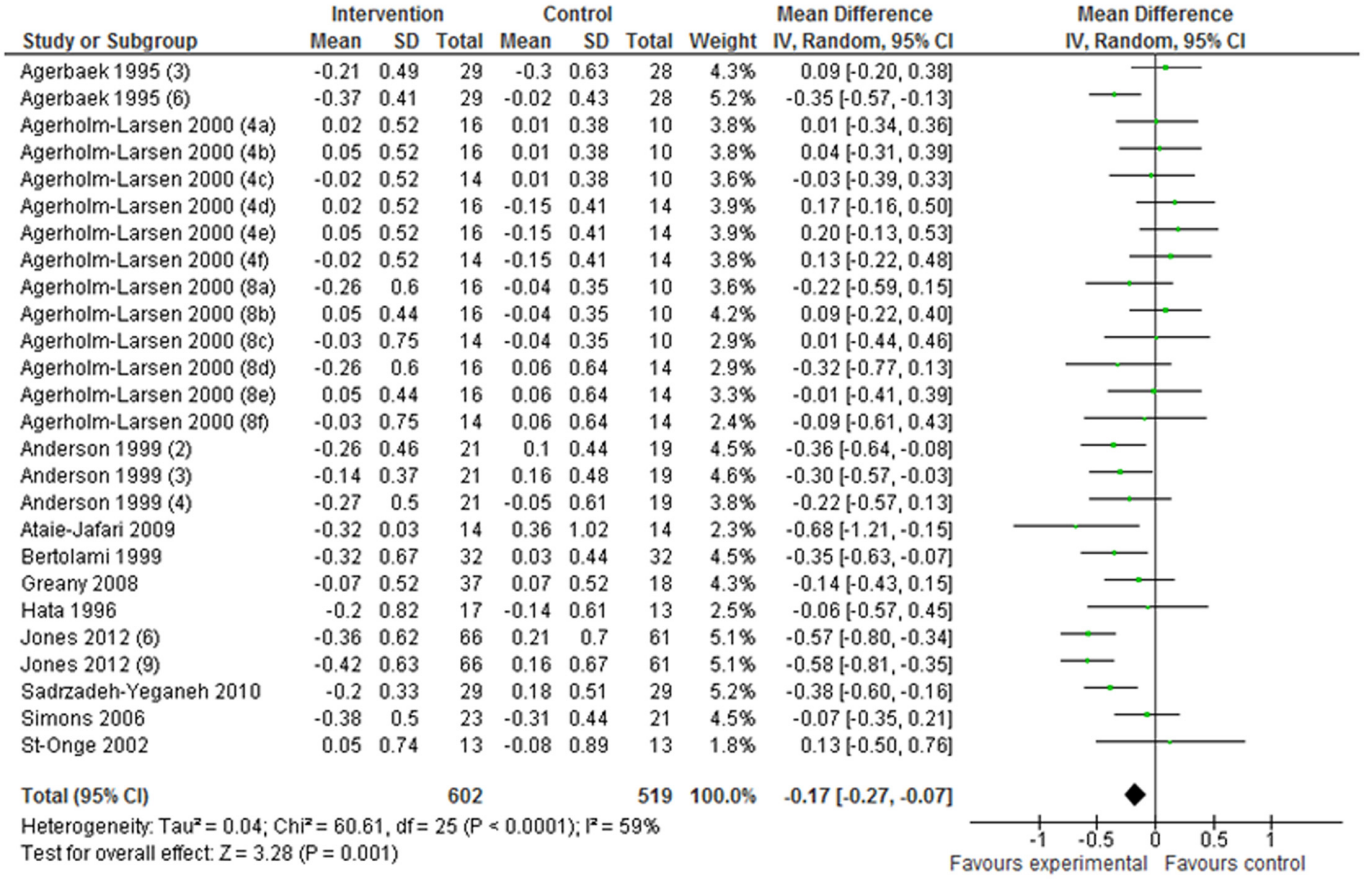
Effects of probiotics on changes in serum TC levels. Values in parentheses indicate intake duration (weeks). a, b, and c in parentheses indicate Gaio, Stra, and StLa vs PP, respectively; d, e, and f in parentheses indicate Gaio, Stra, and StLa vs PY, respectively. PP: placebo pill; PY: placebo yogurt; StLa: *Streptococcus thermophilus* and *Lactobacillus acidophilus*; StLr: *Streptococcus thermophilus* and *Lactobacillus rhamnosus*.

All articles had a Jadad quality score of 3 or higher. Finally, 11 articles (including 26 studies) describing changes (I–C) in TC were selected for the meta-analysis. The mean difference in TC was –0.17 mmol/L (95% CI: –0.27 to –0.07 mmol/L; *P* = 0.001) in the random-effect model analysis. This shows that probiotic interventions significantly decreased serum TC levels (Fig 2).

**Fig 3:**
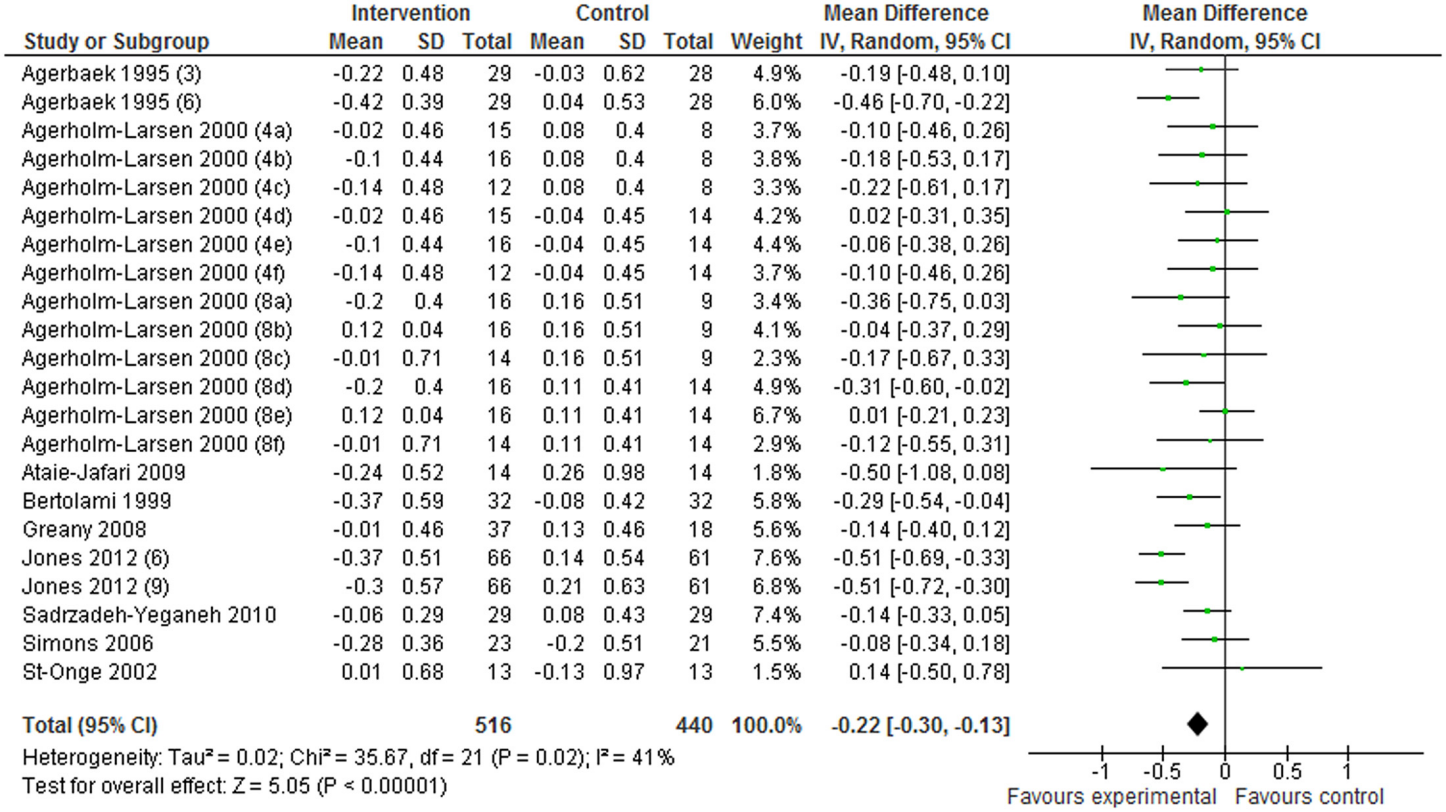
Effects of probiotics on changes in serum LDL-C levels. Values in parentheses indicate intake duration (weeks). a, b, and c in parentheses indicate Gaio, Stra, and StLa vs PP, respectively; d, e, and f in parentheses indicate Gaio, Stra, and StLa vs PY, respectively. PP: placebo pill; PY: placebo yogurt; StLa: *Streptococcus thermophilus* and *Lactobacillus acidophilus*; StLr: *Streptococcus thermophilus* and *Lactobacillus rhamnosus*.

Nine articles (based on 22 studies) describing changes (I–C) in LDL-C were included in the present meta-analysis. The results of the meta-analysis showed that, in comparison with the control groups, probiotic interventions produced a change in LDL-C of –0.22 mmol/L (95% CI: –0.30 to –0.13 mmol/L; *P*<0.00001) in the random-effect model analysis. Probiotic interventions thus significantly decreased serum LDL-C levels (Fig 3).

**Fig 4:**
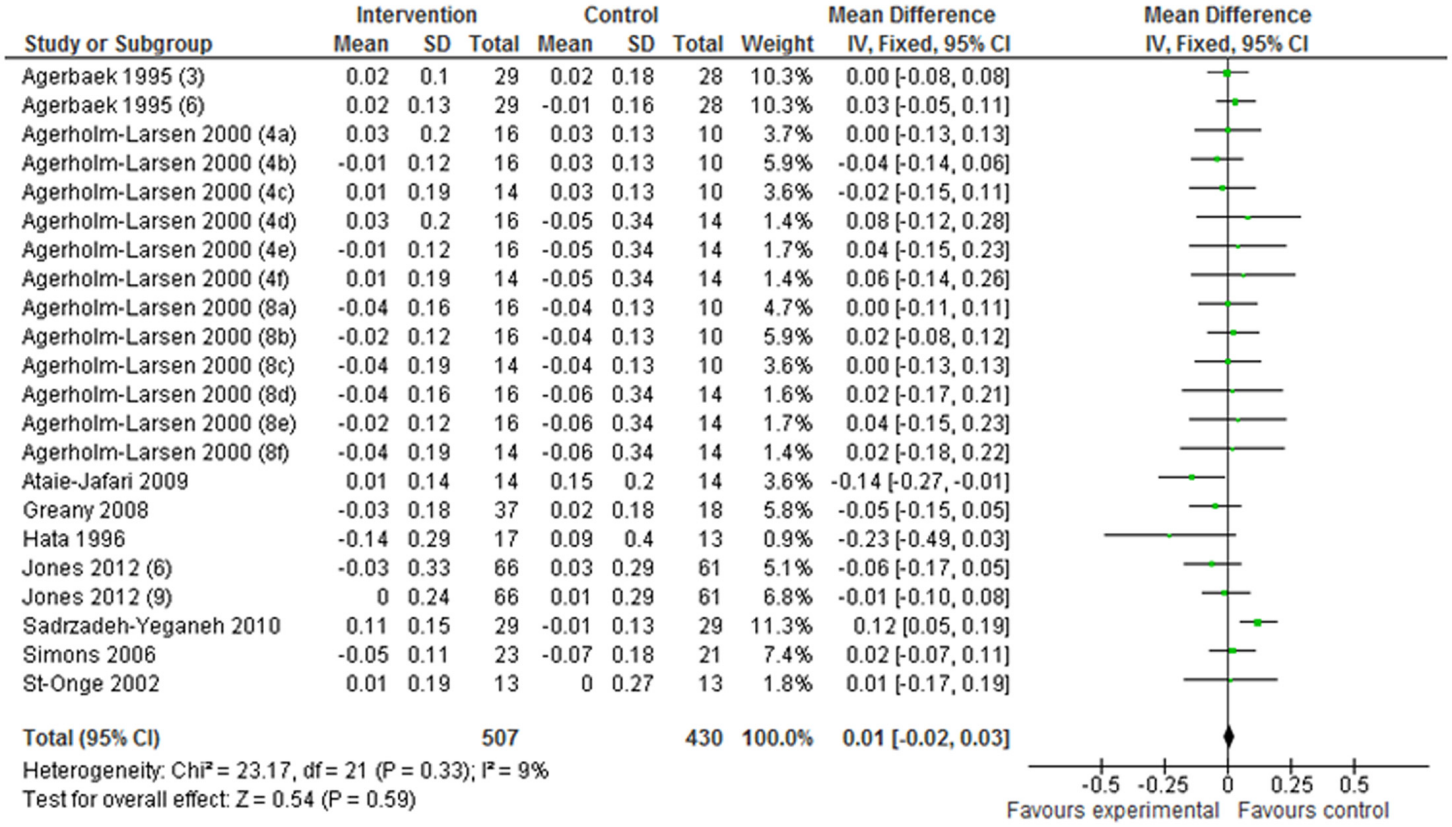
Effects of probiotics on changes in serum HDL-C levels. Values in parentheses indicate intake duration (weeks). a, b, and c in parentheses indicate Gaio, Stra, and StLa vs PP, respectively; d, e, and f in parentheses indicate Gaio, Stra, and StLa vs PY, respectively. PP: placebo pill; PY: placebo yogurt; StLa: *Streptococcus thermophilus* and *Lactobacillus acidophilus*; StLr: *Streptococcus thermophilus* and *Lactobacillus rhamnosus*.

**Fig 5:**
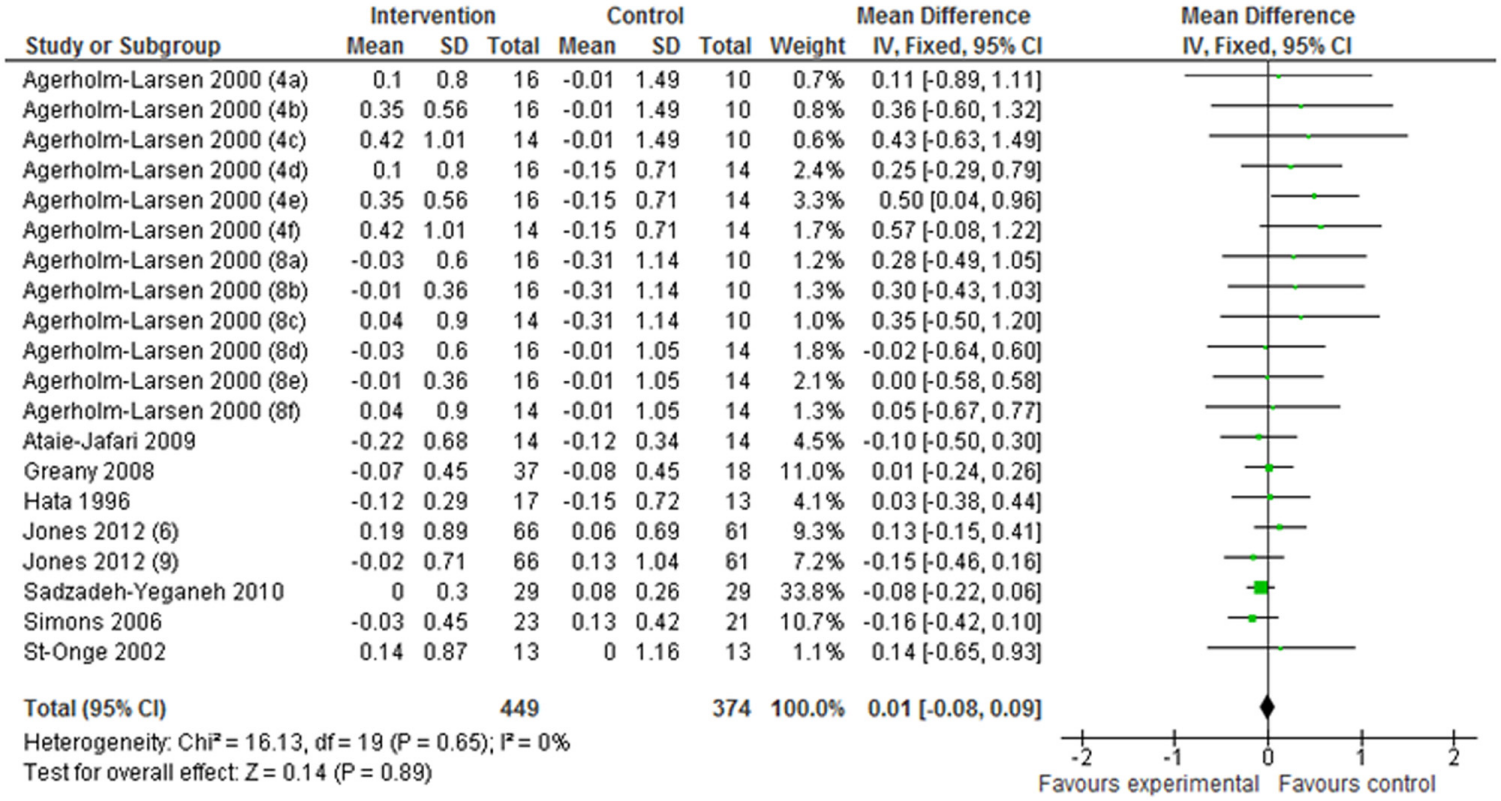
Effects of probiotics on changes in serum TG levels. Values in parentheses indicate intake duration (weeks). a, b, and c in parentheses indicate Gaio, Stra, and StLa vs PP, respectively; d, e, and f in parentheses indicate Gaio, Stra, and StLa vs PY, respectively. PP: placebo pill; PY: placebo yogurt; StLa: *Streptococcus thermophilus* and *Lactobacillus acidophilus*; StLr: *Streptococcus thermophilus* and *Lactobacillus rhamnosus*.

Nine articles (including 22 studies) that described the changes (I–C) in HDL-C were selected for the meta-analysis. The mean difference in HDL-C was 0.01 mmol/L (95% CI: –0.02 to 0.03 mmol/L; *P* = 0.59) in the fixed-effect analysis (Fig 4). Similarly, eight articles (based on 20 studies) that detailed the changes (I–C) in TG levels were subjected to meta-analysis, and the mean difference in TG levels was 0.01 mmol/L (95% CI: –0.08 to 0.09 mmol/L; *P* = 0.89) in the fixed-effect analysis (Fig 5). These data show that HDL-C and TG levels did not differ significantly between the probiotic intervention and control groups.

### Subanalyses

Subanalyses were performed to determine the influence of pre TC and pre LDL-C levels, BMI and age of study participants, duration of probiotic intake, probiotic preparations consumed (“fermented milk product” versus “probiotic preparations”) and bacterial strains on the changes in TC and LDL-C.

Table 2 shows the results of TC subanalyses. Based on the characteristics of participants enrolled, TC decreased significantly in hypercholesterolemic patients (pre TC: ≥5.7 mmol/L and pre LDL-C: >3.6 mmol/L; mean difference: –0.32 mmol/L; 95% CI: –0.45 to –0.18 mmol/L; *P*<0.00001). In terms of probiotic intake, long-term (>4-week) probiotic intervention showed a statistically significant reduction in TC (mean difference: –0.27 mmol/L; 95% CI: –0.39 to –0.15 mmol/L; *P*<0.00001). The consumption of fermented milk products significantly reduced TC (mean difference: –0.22 mmol/L; 95% CI: –0.33 to –0.11 mmol/L; *P* = 0.0001). In addition, decreases in TC levels were greater in the elderly groups (≥45 years of age) than in the younger groups (<45 years of age). In comparison with the mean difference, there were no major differences in the degree of TC reduction between the normal BMI (<25 kg/m^2^) and obese (≥25 kg/m^2^) groups.

**Table 2:** Subanalysis of changes in TC.

		No. of trials(no. of references)	No. of participants(I/C)	MD (95% CI)(mmol/L)	*P* value
Pre TC	Normal (<5.7 mmol/L)	15 (3)	267/204	–0.06 (–0.15, 0.03)	0.18
	Hypercholesterolemic (≥5.7 mmol/L)	11 (7)	335/315	–0.32 (–0.45, –0.18)	<0.00001
Pre LDL-C	Normal (<3.6 mmol/L)	15 (3)	267/204	–0.06 (–0.15, 0.03)	0.18
	Hypercholesterolemic (≥3.6 mmol/L)	11 (7)	335/315	–0.32 (–0.45, –0.18)	<0.00001
BMI	Normal (<25 kg/m^2^)	5 (4)	141/116	–0.23 (–0.35, –0.11)	0.0002
	Obese (≥25 kg/m^2^)	21 (7)	461/403	–0.16 (–0.28, –0.04)	0.01
Age	Young (<45 years of age)	16 (4)	308/247	–0.09 (–0.17, –0.01)	0.03
	Elderly (≥45 years of age)	10 (7)	294/272	–0.37 (–0.47, –0.27)	<0.00001
Intake duration	Short-term (≤4 weeks)	11 (4)	197/170	–0.05 (–0.15, 0.05)	0.36
	Long-term (>4 weeks)	15 (9)	405/349	–0.27 (–0.39, –0.15)	<0.00001

C: control; I: intervention, MD: mean difference, TC: total cholesterol, LDL-C: low-density lipoprotein cholesterol

Table 3 shows the results of subanalyses of LDL-C. Hypercholesterolemic patient groups (pre TC ≥5.7 mmol/L, pre LDL-C >3.6 mmol/L) showed greater decreases in LDL-C levels than normocholesterolemic control groups (pre TC <5.7 mmol/L, pre LDL-C ≤3.6 mmol/L) who consumed probiotics. Long-term (>4-week) probiotic intervention resulted in a statistically significant reduction in LDL-C (mean difference: –0.26 mmol/L; 95% CI: –0.37 to –0.15 mmol/L; *P*<0.00001). In addition, probiotic use was more effective in reducing LDL-C in the elderly (≥45 years of age) than in younger (<45 years old) groups. When comparing probiotic interventions, fermented milk products had similar effects in reducing LDL-C as probiotic preparations. There were no differences in the degree of LDL-C reduction between the normal BMI (<25 kg/m^2^) and obese (≥25 kg/m^2^) groups.

**Table 3:** Subanalysis of changes in LDL-C.

		No. of trials(no. of references)	No. of participants(I/C)	MD (95% CI)(mmol/L)	*P* value
Pre TC	Normal (<5.7 mmol/L)	14 (3)	244/182	–0.12 (–0.20, –0.04)	0.003
	Hypercholesterolemic (≥5.7 mmol/L)	8 (6)	272/258	–0.37 (–0.47, –0.28)	< 0.00001
Pre LDL-C	Normal (≤3.6 mmol/L)	14 (3)	244/182	–0.12 (–0.20, –0.04)	0.003
	Hypercholesterolemic (>3.6 mmol/L)	8 (6)	272/258	–0.37 (–0.47, –0.28)	< 0.00001
BMI	Normal (<25 kg/m^2^)	4 (3)	124/103	–0.22 (–0.34, –0.11)	0.0002
	Obese (≥25 kg/m^2^)	18 (6)	392/337	–0.21 (–0.31, –0.11)	< 0.0001
Age	Young (<45 years of age)	16 (4)	302/238	–0.16 (–0.23, –0.08)	<0.0001
	Elderly (≥45 years of age)	6 (5)	214/202	–0.34 (–0.52, –0.16)	0.0002
Intake duration	Short-term (≤4 weeks)	8 (3)	128/107	–0.11 (–0.23, 0.02)	0.09
	Long-term (>4 weeks)	14 (8)	388/333	–0.26 (–0.37, –0.15)	<0.00001
Preparation	Fermented milk product	10 (6)	208/189	–0.23 (–0.34, –0.13)	<0.00001
	Probiotics	12 (4)	308/251	–0.19 (–0.32, –0.07)	0.003

C: control; I: intervention, MD: mean difference, TC: total cholesterol, LDL-C: low-density lipoprotein cholesterol

Table 4 shows the results of subanalyses by bacterial strain ingested in the reduction of TC and LDL-C compared with placebo. The meta-analysis showed that Gaio (TC: mean difference: –0.14 mmol/L; 95% CI: –0.32 to 0.03 mmol/L; *P* = 0.10, LDL-C: mean difference: –0.26 mmol/L; 95% CI: –0.38 to –0.14 mmol/L; *P*<0.0001) and *L*. *acidophilus* (TC: mean difference: –0.35 mmol/L; 95% CI: –0.48 to –0.22 mmol/L; *P*<0.0001, LDL-C: mean difference: –0.21 mmol/L; 95% CI: –0.49 to 0.07 mmol/L; *P* = 0.14) markedly reduced TC and LDL-C levels, and *Lactobacillus reuteri* NCIMB also markedly reduced TC and LDL-C levels (TC: mean difference: –0.58 mmol/L; 95% CI: –0.74 to –0.41 mmol/L; *P*<0.00001, LDL-C: mean difference: –0.51 mmol/L; 95% CI: –0.65 to –0.37 mmol/L; *P*<0.00001), although there was only a single study of this strain.

**Table 4:** Comparison of bacterial strains between probiotics or FMP and placebo and changes in TC and LDL-C.

	Preparation	Bacterial strain	Reference no.	No. of participants(I/C)	MD (95% CI)(mmol/L)	*P* value
TC						
	FMP	Gaio vs placebo	[20–22]	154/136	–0.14 (–0.32, 0.03)	0.10
		*L*.*acidophilus* vs placebo	[13, 15, 27]	106/100	–0.35 (–0.48, -0.22)	<0.00001
		Sour milk drink vs placebo	[24]	17/13	–0.06 (–0.57, 0.45)	0.82
		Kefir vs milk	[26]	13/13	0.13 (–0.50, 0.76)	0.69
		*L*. *acidophilus*+probiotic vs placebo	[23]	37/18	–0.14 (–0.43, 0.15)	0.35
	Probiotic	*StLa* vs placebo	[21]	64/48	0.09 (–0.08, 0.26)	0.31
		*StLr* vs placebo	[21]	56/48	0.02 (–0.18, 0.22)	0.82
		PCC *L*. *fermentum* vs placebo	[25]	23/21	-0.07 (–0.35, 0.21)	0.62
		*L*. *reuteri* NCIMB 30242 vs placebo	[28]	132/122	–0.58 (–0.74, –0.41)	<0.00001
LDL-C						
	FMP	Gaio vs placebo	[20–22]	152/133	–0.26 (–0.38, -0.14)	<0.0001
		*L*. *acidophilus* vs placebo	[15, 27]	43/43	–0.21 (–0.49, 0.07)	0.14
		Kefir vs milk	[26]	13/13	0.14 (–0.50, 0.78)	0.67
	Probiotic	*StLa* vs Placebo	[21]	64/45	–0.05 (–0.19, 0.10)	0.54
		*StLr* vs Placebo	[21]	52/45	–0.15 (–0.35, 0.06)	0.15
		*L*. *acidophilus*+probiotic vs placebo	[23]	37/18	–0.14 (–0.40, 0.12)	0.29
		PCC *L*. *fermentum* vs placebo	[25]	23/21	-0.08 (–0.34, 0.18)	0.55
		*L*. *reuteri* NCIMB 30242 vs placebo	[28]	132/122	–0.51 (–0.65, –0.37)	<0.00001

TC: total cholesterol; LDL-C: low-density lipoprotein cholesterol; FMP: fermented milk product; *StLa*: *Streptococcus thermophilus* and *Lactobacillus acidophilus*; *StLr*: *Streptococcus thermophilus* and *Lactobacillus rhamnosus*

### Publication bias

**Fig 6:**
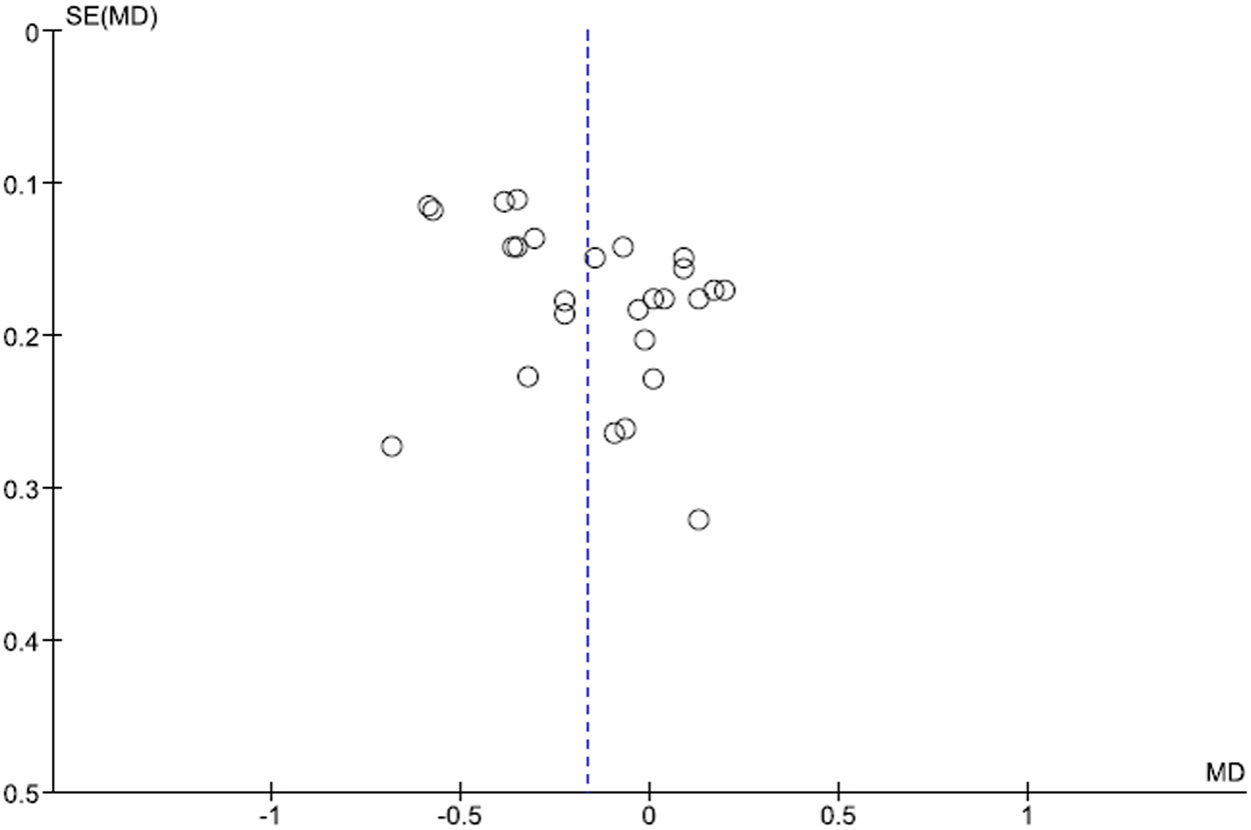
Funnel plots for the results of the 11 articles in the mean difference in the change in the intervention group (I) minus the change in the control group (C) in TC.

**Fig 7:**
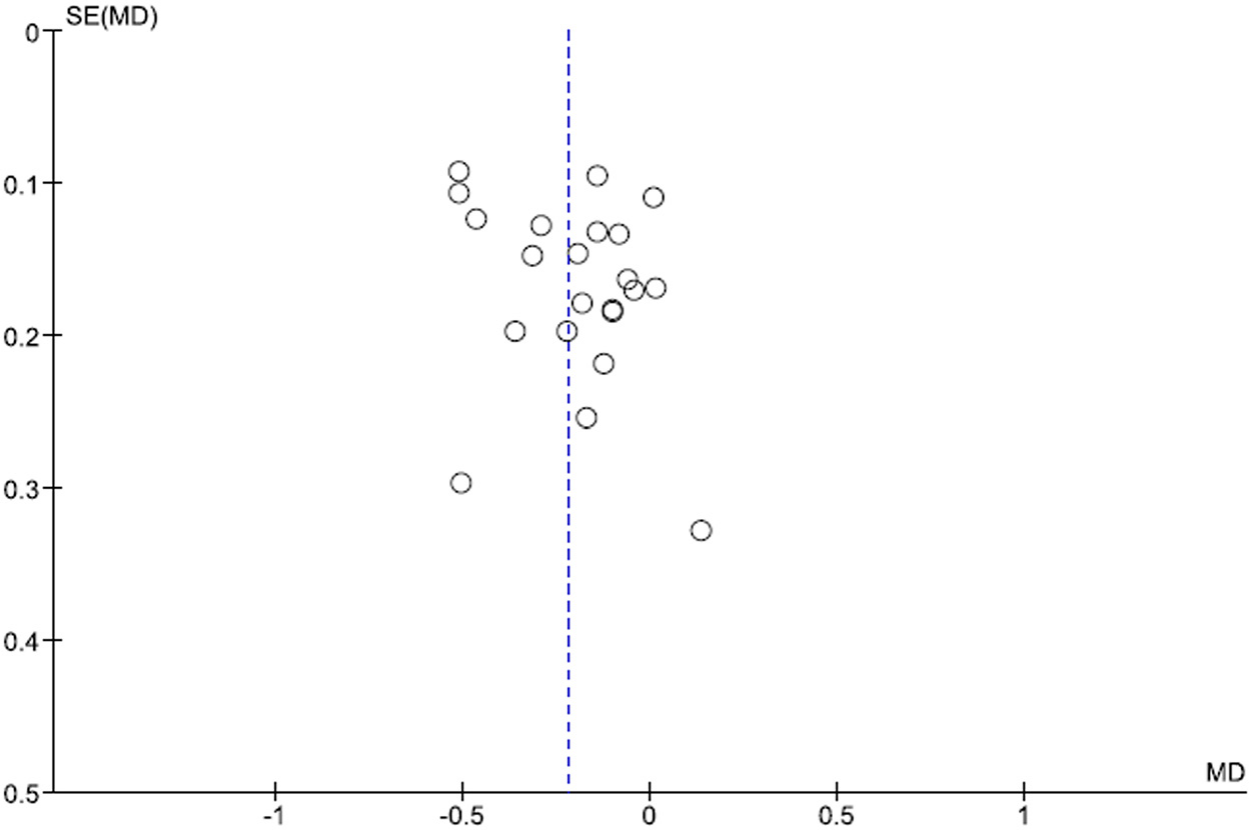
Funnel plots for the results of the 11 articles in the mean difference in the change in the intervention group (I) minus the change in the control group (C) in LDL-C.

The funnel plots for the results of the 11 articles on TC and 9 articles on LDL-C in terms of the mean change in the intervention group (I) minus the change in the control group (C) in TC and LDL-C are shown in Figs 6 and 7, respectively. The results of Begg’s rank correlation method suggested no statistically significant asymmetry in funnel plots for TC and LDL-C analyses, indicating no evidence of substantial publication bias. However, the results of the Egger weighted regression method suggested a statistically significant asymmetry in funnel plots for TC analysis.

## Discussion

The present meta-analysis found statistically significant reductions in TC and LDL-C after probiotic intervention (Figs 1 and 2). Elevated serum TC, LDL-C, and TG concentrations and low HDL-C concentrations are well-established risk factors for CVD [2–4, 29]. Current US national guidelines for CVD risk reduction are primarily focused on strategies to reduce levels of LDL-C, with the most recent focus being on “lower is better” [30]. Based on meta-regression analysis of randomized trials of statins, there was a significant positive relationship between reductions in LDL-C and reductions in the risk for major cardiovascular events [31]. That study suggested that a reduction of 1 mmol/L in LDL-C was associated with a 21% proportional reduction in the incidence of vascular events. Therefore, based on our present results, it is expected that probiotic intervention will lead to an approximately 5% reduction in major cardiovascular events if a linear relationship between reduced LDL-C levels and reduced incidence of vascular events is assumed.

Although several meta-analyses have indicated that probiotic consumption is effective for the improvement of hyperlipidemia, the characteristics of patients who consume probiotics with the most beneficial effects remained unclear. The Japan Atherosclerosis Society Guidelines set diagnostic criteria for dyslipidemia of LDL-C ≥3.6 mmol/L (TC ≥5.7 mmol/L as a reference value), HDL-C ≥1.0 mmol/L, and TG ≥1.7 mmol/L [32]. In those guidelines, an LDL-C level of 3.1–3.6 mmol/L is defined as borderline hypercholesterolemia. On the other hand, the 2013 American College of Cardiology/American Heart Association (ACC/AHA) guidelines on the treatment of serum cholesterol to reduce atherosclerotic cardiovascular risk in adults did not set a target level for cholesterol management and recommended statin therapy in patients with atherosclerotic CVD and LDL-C level of ≥4.9 mmol/L [33]. Although the Japan Atherosclerosis Society Guidelines differ from the 2013 ACC/AHA guidelines, the present meta-analysis used the former to determine the characteristics of serum cholesterol lowering by probiotics. Our results showed that probiotic intervention led to statistically significant reductions in LDL-C and TC levels in hypercholesterolemic patients, which were greater than in normocholesterolemic study participants (Tables 2 and 3). The decrease in LDL-C levels after probiotic intervention in hypercholesterolemic patients would lead to an approximately 8% reduction in major cardiovascular events. Therefore probiotic intervention will have more benefit in patients with hypercholesterolemia than in individuals with normal lipid levels.

Reductions in TC and LDL-C in the elderly were greater than those in younger individuals. This is considered to be due to differences in baseline values, because baseline values of TC and LDL-C in the elderly were higher. In terms of BMI, the mean reductions in TC and LDL-C between normal and obese individuals were almost the same.

In addition, our meta-analysis showed that long-term (>4-week) probiotic intervention resulted in a statistically significant reduction in TC and LDL-C levels (Tables 2 and 3). On the other hand, Agerholm-Larsen et al. [17] found that the duration of probiotic consumption had no significant effect on TC and LDL-C reduction (*P*>0.2) using regression analysis. This difference in findings was likely due to the limited intake duration (4–8 weeks) evaluated by Agerholm-Larsen et al., which is too short to reach optimal statistical power. To the best of our knowledge, this is the first report that long-term (>4-week) probiotic intervention is statistically more effective in decreasing TC and LDL-C levels (Tables 2 and 3), and this information could be useful in CVD risk reduction.

The present meta-analysis included 11 articles, which consisted of 8 [13, 15, 20–22, 24, 26, 27] using fermented milk products and 4 [21, 23, 25, 28] using probiotic capsules as test drugs. One of the 4 articles [23] included both probiotics and prebiotics, i.e., a fructo-oligosaccharide in a rice starch base, as test drugs. The consumption of fermented milk products was associated with statistically significant reductions in TC and LDL-C levels (Tables 2 and 3). Fermented milk products may contain other bioactive components that lower TC and are considered more effective than probiotic preparations, although fermented milk products and probiotic preparations reduce LDL-C to a similar degree.

Species in the *Lactobacillus* and *Bifidobacterium* genera are the most commonly used probiotics, and some studies included in this meta-analysis used *Lactobacillus acidophilus*. Based on *in vitro* experiments, there are several possible mechanisms for the removal of cholesterol from media, such as assimilation of cholesterol during growth by *L*. *acidophilus* [34–36], binding of cholesterol to the cellular surface [34, 35], disruption of cholesterol micelles [34], and deconjugation of bile salt and bile salt hydrolase activity [36]. A recent study has found that *L*. *acidophilu*s reduces cholesterol absorption through the down-regulation of Niemann-Pick C1-like 1 in Caco–2 cells [37]. In the subanalyses by bacterial strain for comparison with placebo in TC and LDL-C reduction, Gaio and *L*. *acidophilus* were shown to reduce TC and LDL-C levels markedly. *L*. *reuteri* NCIMB also markedly reduced TC and LDL-C levels, although it was only included in a single study. This result suggests that the *L*. *acidophilus* strain has greater ability to reduce TC and LDL-C because Gaio contains *L*. *acidophilus*.

This meta-analysis may have certain limitations in the validity and generalizability of results, including methodological limitations. These trials were carried out in various countries including Japan, the EU, the USA, etc., although none of the studies included in the meta-analysis mentioned the ethnicity/race of the clinical trial participants. Although interethnic differences in the cholesterol-lowering effects of probiotics are not known, those effects may vary between ethnic groups, such as Japanese and Caucasians. In addition, the Egger weighted regression method suggested a statistically significant asymmetry in funnel plots for TC analysis, indicating evidence of substantial publication bias, but Begg’s rank correlation method indicated no such evidence. The statistical difference between the two methods seems to be due to the low power in Begg’s rank correlation method.

## Conclusions

This meta-analysis shows that fermented milk products are effective in decreasing TC and LDL-C levels and probiotic preparations are effective in decreasing TC levels. Therefore, probiotic supplementation (fermented milk products and probiotic preparations) could be useful in the primary prevention of hypercholesterolemia and may lead to reductions in risk factors for CVD.

## Supporting Information

S1 Table Prisma checklist. (DOCX)
(DOC)

S2 Table Assessment of the methodological quality of the studies included. (DOCX)
(DOCX)
