# Supplementary material for: Meta-Analysis: Effects of Probiotic Supplementation on Lipid Profiles in Normal to Mildly Hypercholesterolemic Individuals
Source: PLoS One. 2015 Oct 16;10(10):e0139795. doi: 10.1371/journal.pone.0139795 (PMC4608827; doi:10.1371/journal.pone.0139795)
Supplement: S2 Table — (DOCX) (DOCX) [file pone.0139795.s002.docx]

**S2 Table Assessment of the methodological quality of the studies included**

1) single blind, 2) triple blind.
